# Usformer: A small network for left atrium segmentation of 3D LGE MRI

**DOI:** 10.1016/j.heliyon.2024.e28539

**Published:** 2024-03-28

**Authors:** Hui Lin, Santiago López-Tapia, Florian Schiffers, Yunan Wu, Suvai Gunasekaran, Julia Hwang, Dima Bishara, Eugene Kholmovski, Mohammed Elbaz, Rod S. Passman, Daniel Kim, Aggelos K. Katsaggelos

**Affiliations:** a Department of Electrical and Computer Engineering, Northwestern University, Evanston, IL, USA; b Department of Radiology, Northwestern University, Chicago, IL, USA; c Department of Biomedical Engineering, Northwestern University, Evanston, IL, USA; d Department of Medicine, Northwestern University, Chicago, IL, USA; e Department of Biomedical Engineering, Johns Hopkins University, Maryland, USA

**Keywords:** Deep learning, Transformer, Small dataset, Attention, Image segmentation, Left atrium, 3D LGE MRI

## Abstract

Left atrial (LA) fibrosis plays a vital role as a mediator in the progression of atrial fibrillation. 3D late gadolinium-enhancement (LGE) MRI has been proven effective in identifying LA fibrosis. Image analysis of 3D LA LGE involves manual segmentation of the LA wall, which is both lengthy and challenging. Automated segmentation poses challenges owing to the diverse intensities in data from various vendors, the limited contrast between LA and surrounding tissues, and the intricate anatomical structures of the LA. Current approaches relying on 3D networks are computationally intensive since 3D LGE MRIs and the networks are large. Regarding this issue, most researchers came up with two-stage methods: initially identifying the LA center using a scaled-down version of the MRIs and subsequently cropping the full-resolution MRIs around the LA center for final segmentation. We propose a lightweight transformer-based 3D architecture, Usformer, designed to precisely segment LA volume in a single stage, eliminating error propagation associated with suboptimal two-stage training. The transposed attention facilitates capturing the global context in large 3D volumes without significant computation requirements. Usformer outperforms the state-of-the-art supervised learning methods in terms of accuracy and speed. First, with the smallest Hausdorff Distance (HD) and Average Symmetric Surface Distance (ASSD), it achieved a dice score of 93.1% and 92.0% in the 2018 Atrial Segmentation Challenge and our local institutional dataset, respectively. Second, the number of parameters and computation complexity are largely reduced by 2.8x and 3.8x, respectively. Moreover, Usformer does not require a large dataset. When only 16 labeled MRI scans are used for training, Usformer achieves a 92.1% dice score in the challenge dataset. The proposed Usformer delineates the boundaries of the LA wall relatively accurately, which may assist in the clinical translation of LA LGE for planning catheter ablation of atrial fibrillation.

## Highlights

• Usfomer, a transformer-based network is designed for end-to-end segmentation of the left atrium from 3D LGE MRI scans.
• Through the attention mechanism, Usformer acquires overall context across the entire 3D volume.
• The transposed attention largely decreases the network's computational complexity, requiring less data.
• Usfomer shows superior performance in accuracy and computation cost on the challenge and local institutional datasets.

## 1 Introduction

The development of atrial fibrillation (AF) is strongly linked to the presence of left atrial (LA) fibrosis [1], [14]. The accurate assessment of LA fibrosis using 3D late gadolinium-enhanced (LGE) MRI is indispensable for informed clinical diagnosis and treatment planning [2], [14], [20], [30]. However, the current method of labor-intensive manual segmentation introduces noteworthy variability. Therefore, the pursuit of automatic and highly accurate LA segmentation is of great interest for clinical adoption [12], [18], [28]. However, this endeavor encounters challenges due to the intricate nature of LA shapes, patient-specific variations in shapes and sizes, as well as issues of low contrast and background noise [12], [19].

Convolutional neural networks (CNNs) have demonstrated a high level of effectiveness across various applications, like pixel-wise detection of defects with complex and varied shapes [17], [24], [27], [38]. The application of CNNs in LA segmentation is also promising. For example, during the 2018 Atrial Segmentation Challenge, 15 CNN-based methods surpassed the performance of the two traditional atlas-based methods by approximately 7% in dice score [44]. Among these, the methods based on the U-Net [31] model demonstrated the best performance. As a popular self-configuring UNet-based framework, nnU-Net [10] has also demonstrated great performance in the LA segmentation task [34]. The skip connections incorporated into U-Net serve a dual purpose: not only do they recover spatial information for detailed segmentation, but they also effectively address the potential issue of vanishing gradients during training.

**Figure 1:**
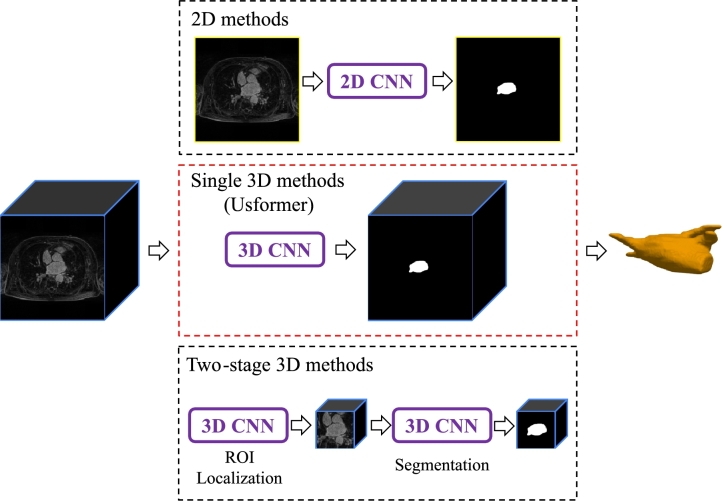
The proposed Usformer belonging to single 3D methods captures the inter-slice correlation not included in the 2D methods and avoids error propagation introduced in two-stage methods.

Approaches for left atrial (LA) segmentation using CNNs can be categorized into three main types: 2D, single 3D, and two-stage 3D methods, as illustrated in Fig. 1. In 2D approaches, each slice of a 3D scan is segmented independently along the out-of-plane axis, and the outcomes from each slice are aggregated to generate the final 3D prediction [3], [35], [40], [43]. For example, GCW-UNet, a 2D U-Net modification developed by Wong et al. [40], obtained a noteworthy dice score of 93.57% in the 2018 Atrial Segmentation Challenge dataset. During the segmentation of individual slices, the model takes in three Gaussian-blurred images, each featuring different degrees of blurring. The inclusion of a channel weight module and Gaussian blurring in GCW-UNet allows for the comprehensive capture of both intricate details and the overall contours of the left atrium (LA). In Bian et al.'s research [3], ResNet [32] was incorporated with dilated convolution and integrated with PSPNet [49]. The inclusion of spatial pyramid pooling merged features at various scales, contributing to improved precision in boundary delineation. Despite the computational efficiency of 2D methods, they might neglect the correlation among adjacent slices in a 3D scan, possibly resulting in inaccuracies in boundary delineation.

On the contrary, 3D techniques involve the direct segmentation of the entire 3D LGE MRI, taking into account the correlation among adjacent slices. Nevertheless, current 3D methodologies exhibit inefficiencies related to both time and memory usage, primarily because of the considerable size of 3D scans. In the 2018 Atrial Segmentation Challenge, the 4th-ranking model is a single 3D CNN, proposed by Vesal et al. [37]. Their approach involved the use of dilated convolution to expand the receptive field and residual connections to gather features from different layers. However, it's noteworthy that this model is the largest in the challenge, containing 104 million parameters—50 times larger than the smallest one.

In an attempt to alleviate the computational and memory demands, many scholars have shifted their focus to implementing two-stage methodologies [13], [42], [45]. Initially, the center of LA is determined by analyzing a down-scaled representation of the LGE MRIs. Subsequently, a fixed zone encompassing this identified center is extracted as the region of interest (ROI). The subsequent step focuses on the segmentation of the LA within this specified ROI. Training two V-Net-based networks with identical architectures but distinct functions, Xia et al. [42] addressed coarse and fine segmentation of the left atrium (LA). The first network's role is to determine the coordinates of the LA center through coarse segmentation, while the second network, utilized in the subsequent stage, focuses on achieving finer segmentation. Rather than utilizing coarse segmentation, Jamart et al. [13] initially implemented a 2D V-net [25] to regress the coordinates of the LA center. Nonetheless, a challenge arises with two-stage methodologies: training both networks simultaneously is intricate, leading to the potential propagation of errors from the initial network to the second one.

Supervised learning methods mentioned earlier often demand a substantial amount of labeled data. But, in some situations, only a limited amount of densely annotated data is available due to the labor-intensive and time-consuming process of delineating the left atrium (LA) boundary. In such cases, semi-supervised learning (SSL)-based methods have been proposed as an alternative to leverage the abundance of unlabeled data to improve LA segmentation [8], [21], [23], [48]. Models in SSL are trained using a combination of limited labeled data and a larger set of unlabeled data. For instance, CA-Net [48] achieves a dice score of 90.09% even when trained with only 16 labeled data and 64 unlabeled data on the 2018 left atrial segmentation challenge. The CA-Net framework incorporates a discriminator that estimates the probability of unlabeled data being treated as labeled data. This mechanism enables the effective utilization of unlabeled data to enhance segmentation performance. However, the accuracy that semi-supervised methods can achieve is much lower than supervised learning methods trained with large-scale datasets, which necessitates a supervised learning method requiring fewer data.

Some previous methods struggle to exploit long-range relations among the pixels in the image and 3D volume. To enlarge the receptive field, CNNs need to increase the kernels' size or the depth of the network, which, however, increases the networks' complexity and requires more training data to avoid overfitting. Different from CNNs, transformer architecture obtains long-range relations with the assistance of self-attention mechanism [5], [22], [41]. In the case of medical image segmentation, transformers have been applied to a wide variety of tasks, such as cell instance segmentation [29] or brain tumor segmentation [39] with promising performance. However, they have huge computation complexity and a large number of parameters. UNeXt [36], a UNet-like architecture using shifted multi-layer perceptions, is proposed to reduce the computation burden and prediction time. But when UNeXt is tailored for the LA segmentation task in a 2D or 3D manner, the accuracy is sacrificed due to shifted multi-layer perceptions.

To address the limitations of the aforementioned methods, we introduce Usformer,^1^ a small 3D transformer-based model aiming to achieve accurate segmentation of LA in just one stage. Within the upper layers, inter-slice correlations are captured by employing 3D convolutions. In the lower layers, the application of transposed attention allows for the extraction of long-range interactions within 3D volumes, with a marked decrease in computational demands compared to regular attention mechanisms. Usformer is validated in the 2018 atrial segmentation challenge [44] and our local institutional NU dataset. It outperforms the state-of-the-art supervised and semi-supervised methods in accuracy, computation complexity, and robustness. Moreover, Usformer does not require a large-scale dataset. The key contributions of this paper can be outlined as follows:

• A postprocessing-free end-to-end network, Usformer, is proposed for accurate left atrium segmentation, which prevents error propagation caused by sub-optimal two-stage training. It has the potential to aid in the clinical translation of 3D LA LGE for planning the ablation of atrial fibrillation.
• A transposed attention module is adopted in Usformer to alleviate the computational burden. Although the standard transformer attention module captures the global context, the burden increases quadratically with the size of the 3D input. Thus through transposed attention, Usformer enables capturing the global context and the correlation among the surrounding slices without increasing the complexity of the model as much.
• Usformer capability is validated in two datasets: the public Atrial Segmentation Challenge Dataset and our local institutional dataset. In both datasets, Uformer outperforms current state-of-the-art methods. Moreover, we demonstrate that Uformer does not require a large dataset. We train it on only 16 densely labeled samples and show that it outperforms other semi-supervised learning methods in accuracy, computation complexity, and robustness.

Subsequent sections are organized as described below: Section 2 provides in-depth insights into the proposed network, offering information about its architecture, attention mechanism, and loss function. Datasets and implement details are described in Section 3. The outcomes of the experiments and corresponding analyses are presented in Section 4. Concluding remarks and future directions are discussed in Section 5.

## 2 Methods

UNet-based methods, while effective for medical image analysis, often struggle to capture global context over the entire image or volume. However, the proposed Usformer addresses this limitation through its transposed attention mechanism. Usformer's architecture and how the transposed attention mechanism works are elaborated upon in this section. A combination of dice loss and binary cross-entropy loss forms the loss function used in Usformer training, which is also described in this section.

### 2.1 The network architecture

**Figure 2:**
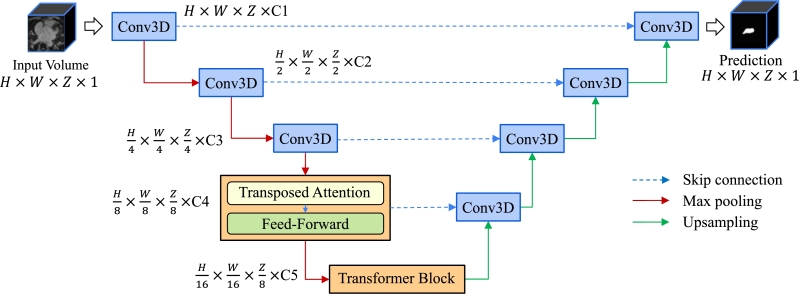
The architecture of Usformer. It is designed for end-to-end left atrium segmentation from 3D LGE MRIs. In the final two stages, the U-Net architecture integrates transformer blocks represented by the orange boxes. The transposed block includes both a transposed attention module (shown in Fig. 3) and a feed-forward network made up of fully connected layers. *H* × *W* × *Z* represents the size of a 3D LGE scan. All feature maps are 3D volumes instead of 2D images. For additional insights into Usformer, please turn to Section 2.

Our proposed model, Usformer, is depicted in Fig. 2. Like the classical U-Net architecture, the encoder and decoder networks of Usformer are on the left and right sides, respectively. Extracting high-level features from the input volume, the encoder network steadily reduces the size of the feature maps while the decoder network progressively reconstructs these features to generate segmentation maps at the original size. Spatial accuracy is improved through the incorporation of skip connections, which establish connections between high-level and low-level features. Despite its merits, the U-Net architecture faces limitations such as a limited receptive field and an inability to capture crucial global information, which plays an essential role in semantic segmentation. Addressing this constraint involves incorporating transformer blocks into the encoder, allowing them to capture the global context through their self-attention mechanism. Therefore, the Usformer encoder is designed with three convolutional stages, followed by two transformer stages. Within each transformer stage, there is one transformer block, succeeded by a convolutional layer and either a max pooling or upsampling layer. Within each transformer block, a transposed attention module and a feed-forward network are present, with the feed-forward network consisting of fully connected layers that typically include non-linear activation functions for introducing non-linearity. As mentioned in Section 2.2, the computation cost grows with the number of input voxels. Thus, to keep the computational cost of the attention down, transformer blocks are put after three convolutional stages to decrease the input size. This architecture also allows each feature vector to encode higher-level information.

A probability map is generated as the segmentation output, illustrating the likelihood of each pixel belonging to the LA. Pixels exceeding a predetermined threshold probability are classified as part of the LA. Threshold values will be explored in Section 3.2.

### 2.2 Attention mechanism

**Figure 3:**
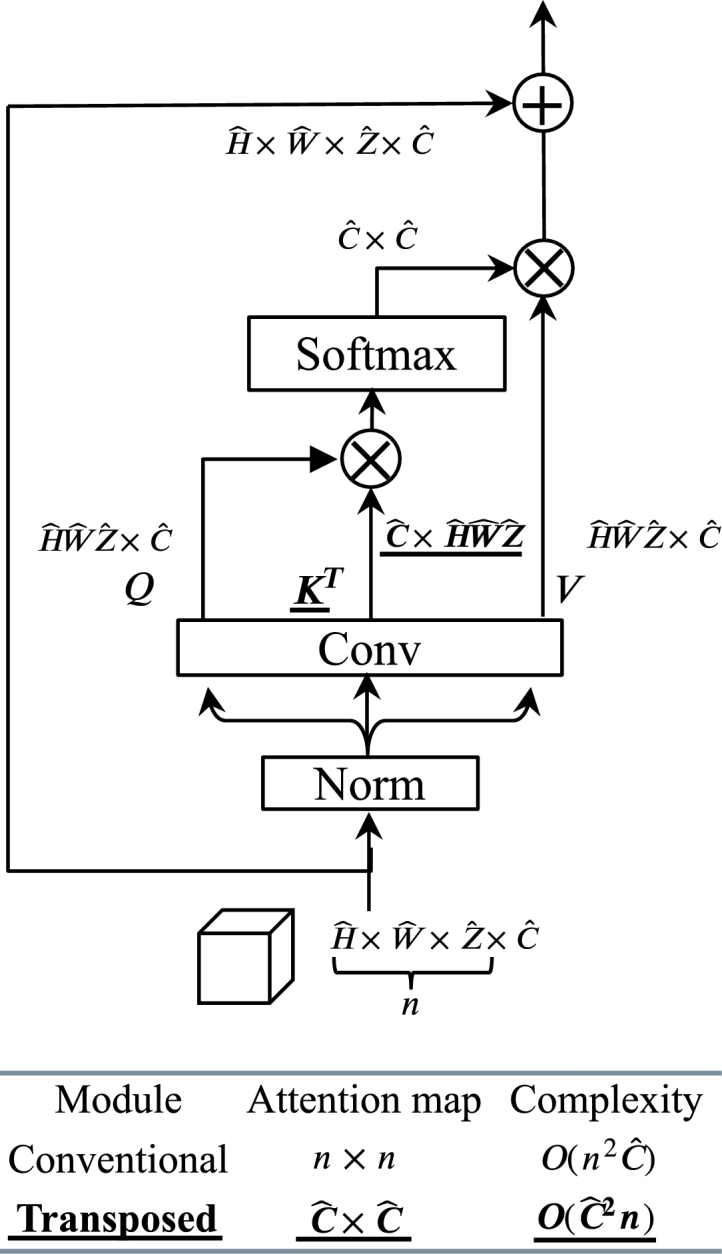
Transformer attention module, where the matrix K is transposed to significantly decrease computation complexity. The output of the transposed attention is calculated by Equation (1). $\overset{ˆ}{H}\times \overset{ˆ}{W}\times \overset{ˆ}{Z}$ represent the input size, and the variable *n* represents the total number of voxels present in the input, which is calculated as $\overset{ˆ}{H}\times \overset{ˆ}{W}\times \overset{ˆ}{Z}$, much larger than the channel number $\overset{ˆ}{C}$. The computation complexity of the transposed module is $O(n^{2}\overset{ˆ}{C})$, much smaller than the conventional module's $O({\overset{ˆ}{C}}^{2}n)$.

The yellow box denotes the transposed attention module [47] in the transformer block. Details are displayed in Fig. 3. Through bias-free convolutional layers, Query (*Q*), Key (*K*), and Value (*V*) are derived from a layer-normalized input of size $\overset{ˆ}{H}\times \overset{ˆ}{W}\times \overset{ˆ}{Z}\times \overset{ˆ}{C}$. The dimensions in the X, Y, and Z directions are denoted by $\overset{ˆ}{H},\overset{ˆ}{W},\overset{ˆ}{Z}$, respectively, with *n* representing the count of input voxels, equal to $\overset{ˆ}{H}\times \overset{ˆ}{W}\times \overset{ˆ}{Z}$. Then, the matrix *K* undergoes transposition to maintain the size of the attention map created by *K* and *Q* at $\overset{ˆ}{C}\times \overset{ˆ}{C}$ instead of n×n. Hence, the computation of the output from transposed attention is as follows:

(1) $$A(V,K,Q)=V\sigma (K^{⊺}Q)$$

In the given equations, $V,K,Q\in R^{n\times \overset{ˆ}{C}}$ serve as three representations of the input within the transposed attention module. The attention scores obtained from the product $K^{⊺}Q$ are transformed into a probability distribution through the softmax function σ(⋅). The softmax function takes the raw scores and converts them into probabilities, ensuring that each score becomes a value between 0 and 1, and the entire set of scores sums to 1. The complexity of computing $K^{⊺}Q$ is in the order of $O({\overset{ˆ}{C}}^{2}n)$. Attention computation in a regular self-attention module [5] follows the equation $A(Q,K,V)=\sigma (KQ^{⊺})V$. With a complexity of $O(n^{2}\overset{ˆ}{C})$, the computation of $KQ^{⊺}$ is considerable. But transposed attention proves to be significantly more computationally efficient, given the constraints $\overset{ˆ}{C}\ll n$ and $O({\overset{ˆ}{C}}^{2}n)\ll O(n^{2}\overset{ˆ}{C})$.

### 2.3 Loss function

Equation (2) serves the purpose of determining the total segmentation loss $L_{seg}$, achieved through a weighted combination of dice loss and binary cross-entropy loss (BCE), as outlined in [4], [6]. The BCE cross-entropy loss $L_{seg}^{BCE}$ assigns equal importance to the loss of all pixels. However, the considerable class imbalance between LA and the background hinders the effective contributions of LA pixels to the training process. In contrast, the dice loss $L_{seg}^{dice}$, being one of the area-based metrics, remains steady irrespective of the background's size, providing a resolution to the class imbalance problem in the LA segmentation dataset [11]. However, relying solely on the dice loss can introduce instability in the training process when the foreground is small, as slight changes can disproportionately impact the dice loss. Thus, it is crucial to include both losses in $L_{seg}$ to ensure a stable and efficient training procedure. $L_{seg}$ is given by:

(2) $$L_{seg}=L_{seg}^{dice}+\lambda L_{seg}^{BCE}=(1-\frac{2Y\cap \overset{ˆ}{Y}}{Y\cup \overset{ˆ}{Y}})-\lambda (Y\text{log}\overset{ˆ}{Y}+(1-Y)\text{log}(1-\overset{ˆ}{Y}))$$

In this expression, *Y* denotes the ground truth, where Y∈{0,1}, and $\overset{ˆ}{Y}$ represents the model output, with $\overset{ˆ}{Y}$ falling in the range of [0, 1]. In this context, 0 denotes the background, while 1 denotes the left atrium (LA). The weight of BCE loss, *λ* will be explored in Section 3.2.

## 3 Experiments

The proposed Usformer is implemented and validated in two datasets, i.e., the commonly used 2018 Atrial Segmentation Challenge dataset [44] and our local institutional NU dataset. Three state-of-the-art supervised learning methods mentioned in Section 1, i.e., the nnU-Net framework [10], UNeXt [36], and TMS-Net [35] are implemented as baselines. The codes of the methods in the 2018 challenge are not publically available, but their published results are compared with Usformer. The 3D dice score, Hausdorff Distance (HD), and Average Symmetric Surface Distance (ASSD) are applied to evaluate the model accuracy. The number of Floating Point Operators (FLOPs) and parameters are applied to evaluate the computation complexity of networks. Moreover, Usformer is also compared with the latest semi-supervised learning methods trained with only a small portion of labeled data. Experimental details and results are discussed in the following sections.

### 3.1 Datasets

Two datasets are utilized to validate the effectiveness of our method: the 2018 Atrial Segmentation Challenge dataset [44] and our local institutional dataset, which are introduced in detail in this subsection.

**2018 Atrial Segmentation Challenge Dataset**^2^ From individuals diagnosed with atrial fibrillation, this dataset contains a total of 154 3D MRI scans. The data were provided by multiple centers but were mostly from The University of Utah. Researchers engaged in the study of LA segmentation commonly utilize this dataset. As listed in Table 1, the image acquisition matrix is 288×288×44 or 320×320×44 pixels with a spatial resolution of 1.25×1.25× 2.5 mm^3^ and then interpreted by a factor of 2 to 576×576×88 or 640×640×88 pixels. The imaging orientation (IJK) is not disclosed on the dataset website. But based on a thorough review of all the slices in the dataset, it appears that the imaging orientation is axial. The challenge's initial training set is randomly divided, with a 4:1 ratio for training and validation. The testing set remains unchanged and corresponds to the original challenge dataset.

**Table 1:** Differences of 3D LGE MRIs in the challenge and NU datasets.

Dataset	Challenge	NU
Imaging orientation	Axial	Coronal (oblique)
Acquisition resolution (mm^3^)	1.25×1.25×2.5	0.75×0.75×2 1.5×1.5×2.2 ......
Acquisition matrix (pixels)	288×288×44 320×320×44	192×192×52 192×192×48 224×224×52

**Our local (Northwestern University [NU]) Dataset** This dataset comprises 178 3D MRI scans provided by Northwestern University. As listed in Table 1, the image acquisition matrix is 192×192×52, 192×192×48 or 224×224×52 pixels with varied spatial resolutions, like 0.75×0.75×2.0 mm^3^, 1.5×1.5×2.2 mm^3^, etc. The imaging orientation (IJK) of the NU dataset is oblique coronal. The original dataset is randomly split into 114 for training, 29 for validation, and 35 for testing.

The manual segmentation of the LA cavity was carried out with consensus by three trained raters for both datasets. This segmentation included structures such as the mitral valve (MV), left atrial appendage (LAA), and pulmonary vein (PV) sleeves. The LA endocardial surface border was meticulously annotated through manual tracing of the PV and LA blood pool. The PV sleeves were limited to a maximum extension of 10 mm from the endocardial surface [26]. Although the criterion for manual segmentation remained the same, the tasks were conducted by different individuals utilizing two different software platforms. The inherent potential for inconsistency in the two datasets is unavoidable.

**Figure 4:**
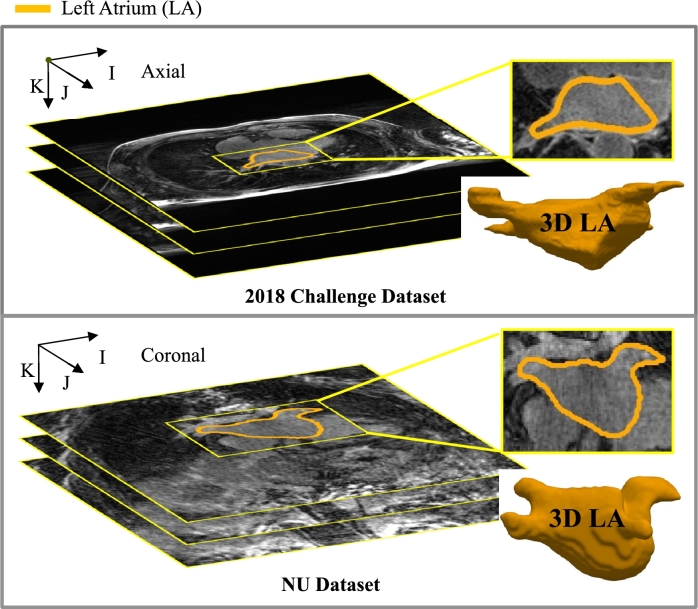
Example 3D LGE MRIs in the challenge and NU datasets with manual segmentations denoted in orange. Each slice of the LGE MRI scans underwent manual segmentation, and the resulting results were aggregated to construct a 3D model of the left atrium. Viewing this figure in color is advised in the printed edition for optimal visualization.

Fig. 4 presents example 3D LGE MRIs in both two datasets with manual segmentations denoted in orange. Manual segmentation was carried out for each LGE MRI, performed slice by slice from the axial view (IJ-plane), and the resulting segmentations were assembled in the K direction to generate the 3D LA geometry. The automated segmentation of LGE scans for the left atrium faces the following challenges:

(1) Class imbalance emerges because the left atrium constitutes a minor portion of the overall volume.

(2) Indistinct boundaries contribute to the challenge of differentiating the left atrium from neighboring tissues.

(3) Reduced image quality poses a challenge in identifying the left atrium. The evaluation of image quality in the 2018 Atrial Segmentation Challenge [44] through the Signal-to-Noise Ratio (SNR) demonstrated that fewer than 15% of the MRI data met the criteria for high quality.

(4) The intricate structure of the anatomy, including slender and lengthy components such as the mitral valve (MV), left atrial appendage (LAA), and pulmonary vein (PV), is a common source of segmentation errors.

(5) The diverse shapes and sizes observed among patients pose a challenge in creating a generalized model for LA segmentation.

(6) Unlike the challenge dataset, the NU dataset presents a greater diversity in imaging orientations and spatial resolution, which is a different challenge.

To mitigate the impact of randomness in the training process, we conducted three random splits for each method. We then calculated the mean and variance of the test results, which are summarized in Table 2, Table 3. By performing multiple splits and reporting the aggregate statistics, we provide a more reliable estimate of the performance of each method and offer insights into the consistency and stability of the results.

**Table 2:** Analysis of the proposed and advanced supervised learning approaches on the challenge dataset's testing set. Displayed in the first four rows are our experiments, while the remaining rows show the results provided by the authors (The codes of rows 5-10 are not publicly accessible). Rows 5-9 are the Top 5 methods in the challenge concerning the 3D dice score, and their results are disclosed in [44].

Method	$N_{t}$/$N_{v}$	Metrics			Computation			
		Dice (%)↑	HD (mm)↓	ASSD (mm) ↓	*N*_*p*_ (M)	FLOPs (G)	*T*_*t*_ (h)	*T*_*p*_ (s/scan)
**Usformer (ours)**	80/20	93.1 ± **1.9**	$\underline{7.2\pm 3.3}$	**0.6 ± 0.2**	5.8	522.9	**5.7**	9.6
nnU-Net [10]	80/20	$\underline{93.1\pm 2.2}$	9.1 ± 4.4	0.8 ± 0.3	16.2	2003.6	7.1	21.2
UNeXt [36]	80/20	90.6 ± 3.4	11.5 ± 5.0	1.1 ± 0.4	15.7	603.3	6.3	16.7
TMS-Net [35]	80/20	91.2 ±2.5	14.1 ± 6.7	1.6 ± 0.6	8	**5.17**	13.3	**5.7**

Xia et al. [42]	80/20	**93.2** ± 2.2	8.9 ± 4.2	$\underline{0.7\pm 0.2}$	21	–	–	–
Huang [9]	80/20	93.1 ± 2.2	8.5 ± 4.1	0.8 ± 0.2	**2**	–	–	–
Bian et al. [3]	70/10	92.6 ± 2.2	9.2 ± 5.3	0.8 ± 0.2	45	–	–	–
Yang et al. [45]	80/20	92.5 ± 2.7	9.8 ± 6.0	0.9 ± 0.3	3	–	–	–
Vesal et al. [37]	64/16	92.5 ± 2.3	9.4 ± 4.7	0.8 ± 0.2	104	–	–	–
Li et al. [15]	100/54	91.9 ± 2.3	**5.9 ± 1.4**	1.0 ± 0.3	5.14	–	–	–

$N_{t}$/$N_{v}$: Number of scans used for training/validation; *HD*: Hausdorff Distance; ASSD: Average Symmetric Surface Distance.

$N_{p}$: Number of parameters; FLOPs: Number of floating point operators; $T_{t}$: Training time; $T_{p}$: Prediction time.

Bold: Best results; Underline: Second-best results.

**Table 3:** Analysis of the proposed and cutting-edge approaches on the NU dataset.

Methods	Dice (%)↑	HD (mm)↓	ASSD (mm) ↓
**Usformer (ours)**	**92.0 ± 3.3**	**10.0 ± 5.7**	**1.0 ± 0.3**
nnU-Net [10]	$\underline{91.6\pm 5.0}$	$\underline{10.9\pm 6.5}$	1.2 ± 0.4
UNeXt [36]	88.5 ± 7.6	15.7 ± 7.0	1.8 ± 0.6
TMS-Net [35]	89.5 ± 4.5	11.1 ± 6.7	$\underline{1.1\pm 0.4}$

*HD*: Hausdorff Distance; ASSD: Average Symmetric Surface Distance.

Bold: Best results; Underline: Second-best results.

### 3.2 Implementation details

3D dice score, Hausdorff Distance (HD), and Average Symmetric Surface Distance (ASSD) [15], [33], [44] are utilized in our paper for model assessment and comparison. Following the 2018 challenge, the dice score is taken as the main metric, and HD and ASSD provide a more comprehensive evaluation of LA segmentation accuracy. The Dice score evaluates the alignment of the segmented LA with the actual LA, while HD and ASSD assess boundary accuracy and spatial dissimilarity. They are formulated using Equations (3), (4), and (5).

Model accuracy is assessed by calculating the average across all scans in the testing set. Moreover, the networks' computational complexity is assessed by considering the number of model parameters and Floating Point Operators (FLOPs).

(3) $$Dice=\frac{2TP}{2TP+FN+FP}$$

In the given equation, *TP*, *TN*, *FN*, and *FP* refer to the count of true positives, true negatives, false negatives, and false positives within the entire volume of each patient.

(4) $$HD=\max\left\{\begin{array}{c} \max_{p\in P}\min_{g\in G}d(p,g),\\ \max_{g\in G}\min_{p\in P}d(p,g) \end{array}\right\}$$

(5) $$ASSD=\frac{\left(\sum_{g\in G}\min_{p\in P}d(p,g)+\sum_{p\in P}\min_{g\in G}d(p,g)\right)}{n_{G}+n_{P}}$$

where *P* and *G* denote surfaces of prediction and ground truth volumes, respectively. p and g are surface voxels in *P* and *G*. d(⋅) represents the distance between two voxels. *n* represents the number of voxels in the corresponding volume.

The Usformer architecture employs 3D convolutions with kernels of size 3×3×3, followed by a max pooling layer with kernels sized 2×2×2. The model's representation ability is progressively enhanced through channel numbers C1, C2, C3, C4, and C5, which are set at 16, 32, 64, 128, and 256, respectively.

In our work, conducting all experiments, we employed a workstation that housed a single NVIDIA A100-PCI GPU card with a memory capacity of 40 GB, a 2.0 GHZ AMD EPYC 7702P CPU, 503 GB of RAM, and running Linux 3.10.0. The Usformer model was trained using the Stochastic Gradient Descent (SGD) optimizer for a total of 200 epochs. Usformer utilized the cosine annealing learning rate schedule, starting with an initial learning rate of 0.001. The cosine annealing learning rate schedule has a smoother learning rate curve, which can contribute to more stable and reliable convergence during training.

To enhance generalizability and prevent overfitting, data augmentation techniques were implemented. A 50% probability was used to apply data augmentation, which included scaling, rotation, and translation to each IJ plane. The I and J axes experienced random selection of the scaling factor, rotation angle, and translation within the intervals (0.5,1.5), $\left(-25^{\circ },25^{\circ }\right)$, and (−10,10) pixels, respectively. Our experimental results demonstrated a 2.1% improvement in the 3D dice score through the application of data augmentation techniques, emphasizing its role in enhancing generalizability and preventing overfitting.

Demonstrating robust performance, our proposed method shows a mere 0.01% difference in the 3D dice score when the threshold (detailed in Section 2.1) is modified within the range of 0.1 to 0.9. The threshold value selected for our work is 0.5.

To determine the weight of BCE loss, *λ*, Usformer was trained with varying *λ* from the value list [0, 0.1, 0.5, 0.9, 1, 10, 100]. The best 3D dice score with Usformer is realized when *λ* is 1 in our analysis. Thus, *λ* is set to 1 in the following experiments.

## 4 Results

### 4.1 Comparative results

**Figure 5:**
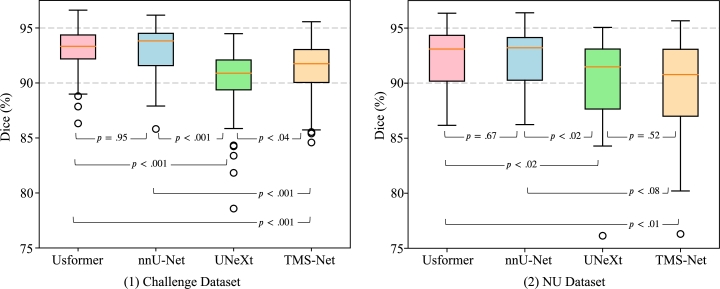
Significant difference analysis between our proposed Usformer and the other three baselines, i.e., nnU-Net [10], UNeXt [36], and TMS-Net [35] concerning the 3D dice score in both challenge and NU datasets.

State-of-the-art supervised learning-based methods to compare with the proposed Usformer include the top 5 methods [3], [9], [37], [42], [45] from the challenge in terms of dice score and the four latest methods unrelated to the challenge [10], [15], [35], [36], as listed in Table 3. Among them, the codes of nnU-Net framework [10], UNeXt [36], and TMS-Net [35] are publicly accessible. Therefore, we implemented these three methods with hyperparameter settings mentioned in their papers on the challenge and NU datasets, listing the results we obtained in Table 2 and Table 3. For a fair comparison, all four models are implemented in the same workstation. Fig. 5 presents a boxplot of their comparative results along with the p-values in the analysis of significant differences. However, it is difficult to replicate the results of the other six methods without public codes. Therefore, we list their published results [15], [44] in Table 2.

By comparing Table 2 and Table 3, it is worth noting that each method exhibits worse performance on the NU dataset, primarily due to the presence of diverse imaging orientations and varying spatial resolutions within the dataset, as listed in Table 1. Despite these challenges, Usformer still achieves promising performance on LA segmentation.

As presented in Table 2 and Table 3, our proposed Usformer outperforms nnU-Net and UNeXt in higher robustness, much fewer parameters, much less number of computation, lower HD and ASSD with similar or higher 3D dice scores. As depicted in Fig. 5, the differences in dice score between Usformer and nnU-Net are not statistically significant (p=0.95, and p=0.67) on the challenge and NU dataset. Although not statistically significant, Usformer achieves higher accuracy 0.4% than nnU-Net on the NU dataset. Compared to UNeXt, Usformer achieves 2.5% higher 3D dice scores on the challenge dataset (p<0.001) and 3.5% higher scores on the NU dataset (p<0.02). Compared to TMS-Net, Usformer achieves 1.9% higher 3D dice scores on the challenge dataset (p<0.001) and 2.5% higher scores on the NU dataset (p<0.01). Moreover, Usformer has the lowest standard deviation among all the methods on both datasets. Two compelling points are that Usformer has a very low number of parameters and computations. In terms of overall parameter count, Usformer (5.8M) is significantly less than nnU-Net (44.7M) and UNeXt (26.5M). The number of Floating Point Operators (FLOPs) is the metric used for assessing the computation. Usformer has the least GFLOPs of 522.9 compared to nnU-Net's 2003.6 and UNeXt's 603.3. We also conducted comparisons of models based on training and prediction times, as listed in Table 2. To enhance accuracy, Usformer makes a slight trade-off in speed when compared to TMS-Net. But Usformer still demonstrates rapid training and prediction capabilities, delivering each prediction within 10 seconds—a quality well-suited for clinical applications.

**Figure 6:**
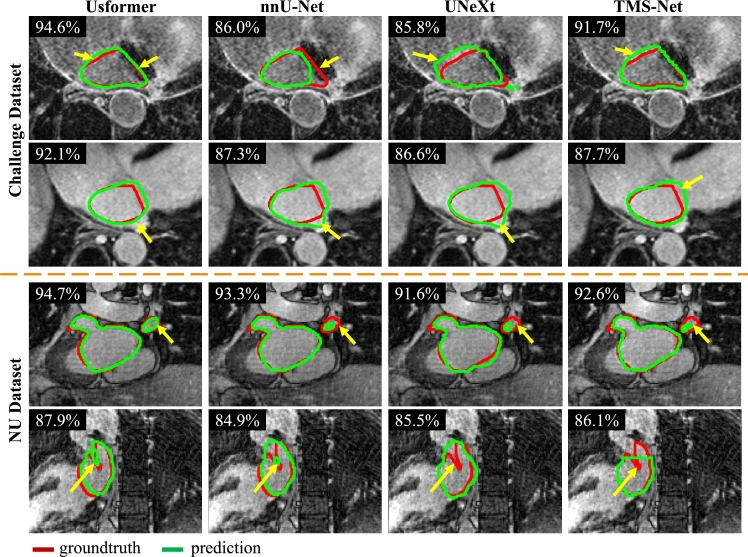
Results of LA segmentation in the axial view by Usformer, nnU-Net [10], UNeXt [36], and TMS-Net [35]. Cases are randomly selected from the challenge and NU datasets, respectively. Each visualization includes the 2D dice score, denoted in the top left corner. Red and green delineate the contours of manual and predicted segmentation. Arrows highlight regions where Usformer exhibits notably superior performance in comparison to the other two baselines. Viewing this figure in color is advised in the printed edition.

Displayed in Fig. 6 are randomly selected examples of LA segmentation results for nnU-Net, UNeXt, TMS-Net, and Usformer. The challenge dataset is depicted in the first two rows, while the NU dataset is illustrated in the last two rows. Our proposed approach exhibits a high level of precision in delineating LA segments, not only at small sizes (highlighted by the yellow arrow in the third row), but also with complex shapes (highlighted by the yellow arrow in the last row). Even though the boundary between LA and RA is unclear (highlighted by the yellow arrow in the first row), Usformer delineates the boundary accurately. Furthermore, Our approach surpasses nnU-Net, UNeXt, and TMS-Net, achieving markedly higher 2D dice scores. The segmentation outcomes exhibit significantly improved proximity to the manual segmentation, as highlighted by all the yellow arrows.

Usformer's efficiency is improved with the integration of the transposed attention module, which reduces computational complexity and facilitates the understanding of global information. This guarantees Usformer's potential for efficient, accurate, and robust LA segmentation, as demonstrated in Table 2, Table 3, and Fig. 6.

Notably, as for the first two cases in Fig. 6, Usformer's improvement in 2D dice is larger than the improvement in 3D dice. There are two main reasons. First, a model's performance varies across different individual patients and slices, as shown in Figure 5, Figure 6. In some cases, Usformer performs better than other models, and vice versa. Second, the 2D dice score tends to change more than the 3D dice score with the same number of pixels' changes since the denominator of the 2D dice is much smaller. Therefore, both in the 2018 challenge and our work, the average of 3D dice is utilized as one of the metrics of accuracy rather than the average 2D Dice.

### 4.2 Error analysis

**Figure 7:**
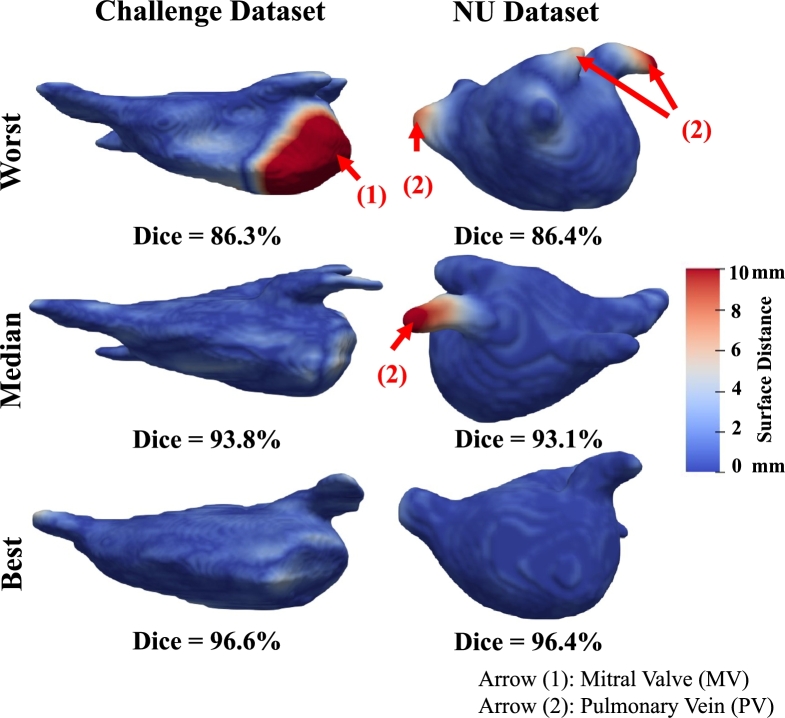
Three-dimensional representation of the best, median, and worst left atrium segmentation implemented by our method regarding the 3D dice score. The first and second columns are from the challenge and NU datasets, respectively. Distance from the manual segmentation to the prediction is indicated by the color of the surface. For improved visualization, the surface distances are rescaled within the range of 0 to 10 mm. Arrows (1) and (2) highlight the errors in MV and PV, respectively. Viewing this figure in color is advised in the printed edition.

**Figure 8:**
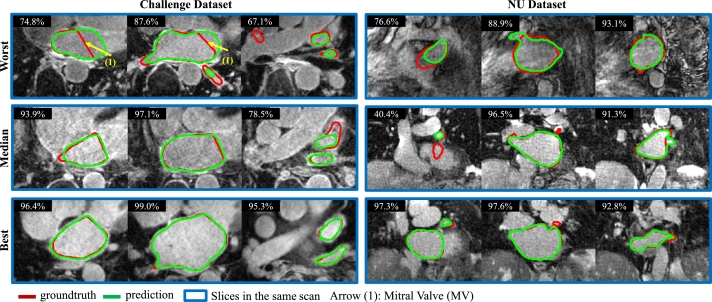
Results of LA segmentation in the axial view by our Usformer on the challenge and NU datasets. The three rows display cases with the worst, median, and best performances by Usformer, as measured by the 3D dice score. Three slices of each example case are presented. The 2D dice score is indicated in the top left corner of each visualization. Red and green delineate the contours of manual and predicted segmentation. Viewing this figure in color is advised in the printed edition.

For each dataset, three cases are selected from each testing set for 3D and 2D visualization, representing the worst, median, and best performances in terms of the proposed method's 3D dice score, as shown in Figure 7, Figure 8. Fig. 7 visualizes the surface distance between manual segmentation and prediction by our proposed Usformer. LA segmentation results in the axial perspective are depicted in Fig. 8.

Figure 7, Figure 8 indicate that our proposed method exhibits favorable outcomes in left atrium segmentation, even in the face of substantial variations in the shapes observed across patients. The LA shapes are complex, but the overall predictions demonstrate a smooth and accurate outcome. With respect to the surface distance, the error is small and lies within the tolerance range. The proposed method showcases its proficiency in outlining left atrial (LA) segments in the last two rows of Fig. 8, effectively handling complex shapes and challenges arising from low contrast with the surroundings.

As shown in Figure 7, Figure 8, the main errors are on the MV (highlighted by arrow (1)) and the PV (highlighted by arrow (2)). The errors in the MV can be attributed to the unclear boundary between LA and LV and the flat shape labeled by observers. As pointed out by arrows (1) in Fig. 8, the mitral valve (MV) identified by the observers as a flat plane was predicted by the proposed method as a circle, resulting in numerous false positives. The area containing errors has poor contrast, and observers may segment the region with significant variability, leading to confusion for the network. The errors observed in the PV are primarily attributed to its elongated, slender, and diverse shapes. Observers might segment the PVs with varying lengths, contributing to confusion for the network.

### 4.3 Dataset scale

**Figure 9:**
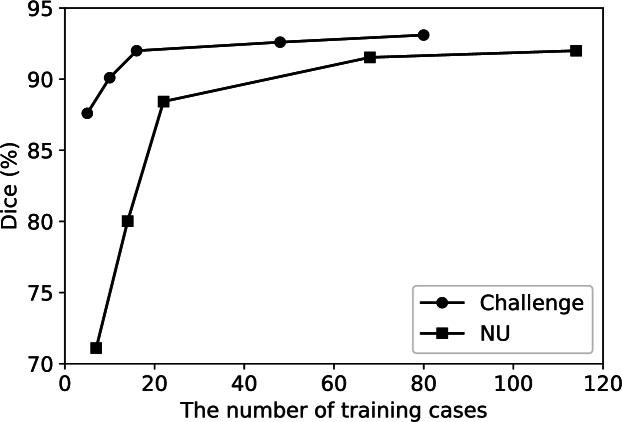
The performance of Usformer trained with different numbers of cases in the challenge and NU datasets.

Usformer was trained using different amounts of training cases and tested on the same testing set to explore how the amount of training samples affects the performance of LA segmentation. Fig. 9 presents the trends on the challenge and NU datasets. A certain number of cases from the training set was randomly selected for training each time. We repeated each experiment three times and reported the average of three tested results.

Usformer has good performance even though it just used 16 training cases. As shown in Fig. 9, Usformer reached a dice score of 92.1% even though it was trained only with 16 labeled scans in the challenge dataset. Compared with the challenge dataset, Usformer requires a larger scale in dealing with the NU dataset due to its more varied imaging orientations and spacings. To reach a dice score of 91.6%, Usformer requires 68 labeled scans from the NU dataset.

The latest semi-supervised methods only use the training set of the challenge data and divide it into two sets, 80 scans for training and 20 for testing. They were trained on the 16 labeled and 64 unlabeled scans from their training set. For comparison purposes, we trained Usformer using 16 scans randomly selected from their training set and tested on the same testing set they disclosed online. We repeated the experiment three times and took the average of three tested results. As listed in Table 4, it is obvious that Usformer outperforms the latest semi-supervised methods by 1.1% in terms of dice score while only 16 annotations are available for training.

**Table 4:** Comparative results with the latest semi-supervised learning methods while only 16 annotations are available for training. All methods are tested in the same set. The authors' disclosed results are listed in the table.

Methods	Dice (%)
**Usformer**	**92.4**
CC-Net [8]	91.3
CA-Net [48]	90.1
UA-MT [46]	88.9
SCC [21]	89.8
SASSNet [16]	89.3
LG-ER-MT [7]	89.6
DTC [23]	89.4

Bold: Best result.

Underline: Second-best result.

## 5 Discussion and conclusions

Accurate segmentation of the left atrium (LA) is crucial for the assessment of LA fibrosis, aiding in informed clinical diagnosis and treatment planning. The study introduces Usformer, a network characterized by its small size, speed, and accuracy in the left atrium (LA) segmentation. Three significant contributions are outlined. Firstly, the implementation of an end-to-end framework eliminates error propagation inherent in two-stage methods. Secondly, the incorporation of transposed attention within Transformer blocks enables the learning of long-range dependencies among voxels in large 3D volumes without bringing a high computational cost. As detailed in Section 1, the 3D CNN-based method proposed by Vesal et al. [37] carries a substantial computational and memory burden, amounting to 18 times the parameter count of our Usformer. Lastly, the reduced complexity of our model allows it to train with a reduced dataset while still achieving promising performance in LA segmentation (see Section 4).

Our method proves effective even in the presence of challenging image quality in LGE MRI, demonstrating promising outcomes on both the NU dataset and the public 2018 Atrial Segmentation Challenge set, achieving average 3D dice scores of 93.1% and 92.0%, respectively. The number of parameters and computation complexity of Usformer, respectively, are reduced by 2.8x and 3.8x over the state-of-the-art nnU-Net. Moreover, Usformer outperforms the latest semi-supervised learning method, CA-Net, by 2.3% in terms of dice score when trained with only 16 labeled MRI scans.

It is unclear how well the presented method can adapt to modalities beyond LGE. To investigate the model's generalization capabilities, we plan to expand our dataset by collecting samples from various modalities, machines, and centers. Moreover, to gain a more complete insight into LA anatomy, future research could investigate the combination of various imaging modalities, including computed tomography or alternative MRI sequences.

To conclude, the proposed small Usformer delineates the left atrium in LGE MRI scans with high accuracy and low computation memory. It introduces a versatile and viable choice, minimizing the expenses associated with manual segmentation. The proposed network is expected to demonstrate success in various segmentation challenges.

## CRediT authorship contribution statement

**Hui Lin:** Supervision, Resources, Project administration, Writing – review & editing, Writing – original draft, Visualization, Validation, Software, Methodology, Investigation, Formal analysis, Data curation, Conceptualization. **Santiago López-Tapia:** Writing – review & editing, Supervision, Software, Methodology, Formal analysis, Conceptualization. **Florian Schiffers:** Writing – review & editing, Supervision, Formal analysis, Conceptualization. **Yunan Wu:** Writing – review & editing. **Suvai Gunasekaran:** Resources, Data curation. **Julia Hwang:** Resources, Data curation. **Dima Bishara:** Resources, Data curation. **Eugene Kholmovski:** Resources, Data curation. **Mohammed Elbaz:** Funding acquisition. **Rod S. Passman:** Resources, Funding acquisition. **Daniel Kim:** Writing – review & editing, Supervision, Resources, Project administration, Funding acquisition. **Aggelos K. Katsaggelos:** Writing – review & editing, Supervision, Resources, Project administration, Investigation, Funding acquisition, Conceptualization.

## Declaration of Competing Interest

The authors declare that they have no known competing financial interests or personal relationships that could have appeared to influence the work reported in this paper.

## Appendix A Supplementary material

Video 1 Median segmentation performance achieved by Usformer on the challenge dataset. Video 1

Video 2 Median segmentation performance achieved by Usformer on the NU dataset. Video 2

## Footnotes

1 The code is available at https://github.com/HuiLin0220/Usformer.git.

2 https://www.cardiacatlas.org/atriaseg2018-challenge/atria-seg-data/.
